# Minimizing Delay and Power Consumption at the Edge

**DOI:** 10.3390/s25020502

**Published:** 2025-01-16

**Authors:** Erol Gelenbe

**Affiliations:** 1 Institute of Theoretical & Applied Informatics, Polish Academy of Sciences (IITiS-PAN), 44-100 Gliwice, Poland; seg@iitis.pl; 2 Université Côte d’Azur, CNRS I3S, 06107 Nice, France; 3 Department of Engineering, King’s College, London SE1 8WA, UK

**Keywords:** edge computing, sensor networks, edge computing, latency minimization, reducing energy consumption, G-networks, analytical solution

## Abstract

Edge computing systems must offer low latency at low cost and low power consumption for sensors and other applications, including the IoT, smart vehicles, smart homes, and 6G. Thus, substantial research has been conducted to identify optimum task allocation schemes in this context using non-linear optimization, machine learning, and market-based algorithms. Prior work has mainly focused on two methodologies: (i) formulating non-linear optimizations that lead to NP-hard problems, which are processed via heuristics, and (ii) using AI-based formulations, such as reinforcement learning, that are then tested with simulations. These prior approaches have two shortcomings: (a) there is no guarantee that optimum solutions are achieved, and (b) they do not provide an explicit formula for the fraction of tasks that are allocated to the different servers to achieve a specified optimum. This paper offers a radically different and mathematically based principled method that explicitly computes the optimum fraction of jobs that should be allocated to the different servers to (1) minimize the average latency (delay) of the jobs that are allocated to the edge servers and (2) minimize the average energy consumption of these jobs at the set of edge servers. These results are obtained with a mathematical model of a multiple-server edge system that is managed by a task distribution platform, whose equations are derived and solved using methods from stochastic processes. This approach has low computational cost and provides simple linear complexity formulas to compute the fraction of tasks that should be assigned to the different servers to achieve minimum latency and minimum energy consumption.

## 1. Introduction

The advent of the Internet of Things (IoT) and related technologies, such as smart homes, smart vehicles, 5th generation (5G) networks, and beyond 5G, increase the need for high throughput, low task delays, and low energy consumption through the development of systems that provide computing and communication services at the edge [1,2]. While radio access networks (RANs) and mobile base stations can massively increase the bandwidth and throughput that is offered to end users through these technologies, applications are also being moved from cloud computing platforms to the edge of the Internet [3,4,5] to achieve high throughput with low latency and lower energy consumption [6,7]. Motivated by these developments, much research has been conducted to allocate tasks in edge systems in a manner that attempts to minimize latency and energy consumption using non-linear optimization techniques [8,9] leading to NP-hard problems, which are processed with various heuristics and approximations, or with AI-based approaches [10,11], such as reinforcement learning. These previous approaches have some shortcomings: (a) there is no guarantee that optimum solutions are achieved, and (b) they do not provide a clear indication of the fraction of tasks that should be allocated to the different servers to achieve a specified optimum. Also, the parameters that are used by these methods must be measured and updated to construct the required algorithms; the methods are computationally costly, with additional overhead and energy consumption required for lightweight edge systems. In addition, these approaches do not provide insight into the key parameters, such as the task allocation rates or proportion of tasks that should be sent to different servers, to guarantee that the system will operate at or near its optimum point.

Thus, this paper proposes a radically different, mathematically based, and principled approach that explicitly computes the optimum fraction of jobs that should be allocated to the different servers to either (1) minimize the average latency (delay) of the jobs that are allocated to the edge servers or (2) minimize the average energy consumption of the jobs that use the edge servers. To achieve these objectives, this paper develops a mathematical model of a multiple-server system that is managed by a task dispatching platform (DP). The model equations are derived and solved using methods from stochastic processes. We then use this theoretical framework to explicitly derive the optimum workload distribution that minimizes latency. The paper then uses a similar approach to derive an explicit expression for the share of workload that should be allocated to each edge server that minimizes the system’s additional energy consumption per task. The analytical approach we develop has a low computational cost and provides detailed insight into the fraction of tasks that are allocated to the different servers to achieve minimum latency and minimum energy consumption.

### 1.1. The Main Results Presented in This Paper

After the review of related work on the design of task-dispatching algorithms that optimize edge performance discussed in Section 1.2, the architecture of an edge system that includes a decision platform (DP) that dispatches incoming external tasks to a set of *n* servers is presented in Section 2. Then, the notation and symbols used in the paper are summarized in Section 2.1. All the proofs related to the theoretical developments in the paper are presented in detail in separate appendix sections that are clearly linked to the sections where the results are presented.

A novel mathematical model of an edge system composed of the DP that sends tasks to *n* servers is presented in Section 3. The Key Product Form Result for this model is stated and proved in Theorem 1, and Lemma 1 shows that its solution accounts for the processing of all the tasks that enter the system. Then, in Section 4, we show how the decision variables $C_{i},\;1\leq i\leq n$, which combine the requests from the *n* servers with the task assignment decisions that are made by the DP to each server, affect the average latency of externally arriving tasks at the DP.

Then, Section 5 derives the task allocation policy that minimizes the average response time for all tasks being processed at the *n* servers in the system. Section 6 discusses the power consumption of edge servers based on power measurements that were made on NUCs and other processors, and we derive a policy that depends on the known parameters of each server to share the tasks between servers to guarantee that the average **energy consumption** for incoming tasks at the edge is minimized.

Finally, Section 7 provides conclusions and directions for further work.

### 1.2. Related Work

There has been considerable work on the design of algorithms for distributed system management and task distribution to reduce response times for tasks and maximize data transfer throughputs [12,13]. Real-time techniques have been developed to this effect [14], and various heuristics have often been tested in simulated environments to balance load and reduce response times [15,16]. Energy consumption has been of increasing concern over the last decade because of the steady increase observed over this period in the power consumption of ICT [5,17,18].

Recent research in this area has been primarily motivated by the need for low-cost distributed systems that offer computation and data-intensive applications close to the network edge to achieve low latency [19] for mobile technologies, the IoT, and smart vehicles [20]. Another motivation is the need for distributed computing facilities that locally serve small-scale applications, such as smart homes [21], and in some recent work [22], a system was considered where tasks that arrive at an edge server are either directly executed there or off-loaded to a different server.

As early as the 1990s, the research community proposed AI-based dynamic network management techniques [23,24,25,26] that were later facilitated by the introduction of Software Defined Networks [27] to achieve improvements in network performance and security [28,29]. Attempts have been made to use reinforcement learning or, more broadly, machine learning [30,31,32] as a tool to reduce latency and achieve power savings for tasks that are sensitive to the “quality of service” [33]. Other work has integrated security needs by managing tasks and flows of data so that insecure servers and networks may be dynamically avoided [34,35]. Market-based bidding techniques and games to design low computational cost algorithms that have been shown to offer fast solutions at low cost during simulations [36,37]. Some practical experiments have tested AI in distributed edge systems using Software Defined Networks to reduce latency and improve power consumption [38]. Since edge systems often fulfill multiple functions and support a variety of users, the resulting optimization problems are often NP-hard, and heuristic approximations are often investigated [39].

## 2. System Description

We consider an edge distributed computing system composed of a Dispatching Platform (DP) that resides on a separate server with *n* machines or servers, $S_{1},\ldots ,S_{n}$, that together form a cluster that is accessible through the Internet. Each $S_{i}$ receives local tasks to execute, as well as tasks that are allocated to it by the DP. External tasks to be executed by the edge system are received by the DP and assigned to the servers based on requests from the servers.

**Figure 1:**
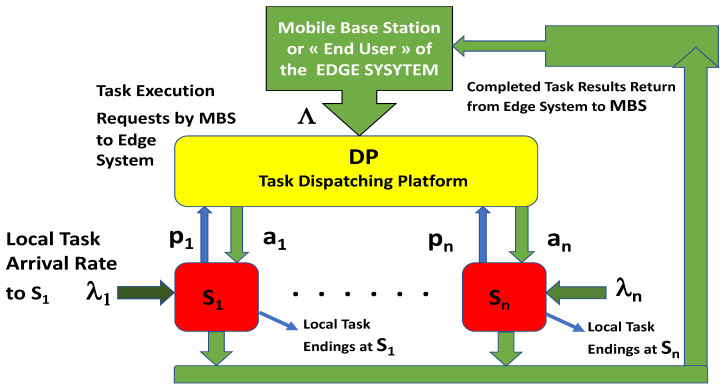
Architecture of an edge system that allocates incoming tasks to a set of locally connected servers for edge computing [40]. It is composed of a Dispatching Platform (DP) that dynamically exploits the *n* distinct servers’ available capacity to allocate tasks to minimize average task delay or to minimize total power consumption. Each server has its own incoming local flow of tasks, and each server requests and receives tasks from the DP.

The base station or external user shown in Figure 1 sends tasks to the DP, where they are stored in an input queue as they wait for task requests from the *n* edge servers.

- When any $S_{i}$ completes the current task that it is executing, it makes a task request from the DP with probability $1\leq p_{i}\leq 1$. If the DP task input queue is empty, then the request is simply rejected by the DP. If the DP task input queue contains at least one task, then the DP assigns the task to $S_{i}$ with probability $0\leq a_{i}\leq 1$.
- Thus, when $S_{i}$ terminates an ongoing task, a task from the incoming pool is dispatched by the DP to $S_{i}$ with probability $C_{i}=p_{i}a_{i}$, **provided that the input queue at the DP is not empty**. If the DP queue is empty, obviously, no task can be sent. This is equivalent to assuming that when a server $S_{i}$ informs the DP that it has terminated a task, then the DP allocates a task to $S_{i}$ with probability $0\leq C_{i}\leq 1$ if the DP has a task waiting at its input. If there are no tasks waiting at the DP, then the request from $S_{i}$ is rejected.
- Note that task endings at the different servers occur asynchronously with each other, and the decision of the DP is simply to send or not to send a new task to $S_{i}$.
- Thus, each server has a queue of tasks, some of which have been sent by the DP and others are local tasks that it receives and executes.

External tasks arrive at the DP at a rate Λ>0 (tasks per second), while each $S_{i}$ receives “locally generated tasks”, e.g., from its local owner or user or as part of its operating system at the following rate:

(1) $$\lambda_{i}\geq 0,\;\lambda =\sum_{i=1}^{n}\lambda_{i}\;.$$

The average execution time of each task at $S_{i}$ is denoted by $\mu_{i}^{-1}$.

The DP’s objective is to minimize the total average waiting time at the DP and the average response time at all the *n* servers. However, it also aims to reduce the overall energy consumption of the system. On the other hand, each $S_{i}$ must execute all the tasks it has received locally, as well as those that it has requested from the DP and that the DP has allocated to it. The $S_{i}$ may need to generate income from the external tasks it receives from the DP. On the other hand, it also needs to provide low latency (i.e., low response time) for all the tasks it receives. The DP, as well as all $S_{i}$, also aims to keep the overall average energy consumption as low as possible because of the cost of the energy and to achieve greater sustainability.

### 2.1. Summary of Notation and Symbols and Abbreviations

In this sub-section, we present and define all the **symbols that are used throughout this paper**.

- t≥0 is the real-valued time variable.
- DP is the task dispatching platform that transfers tasks from the end users to the servers.
- $S_{i}$ denotes a server that receives tasks assigned by the DP, as well as “locally generated tasks”, e.g., from its local owner or user or as part of its operating system.
- Λ>0 is the rate of arrival of external tasks to the DP.
- $\lambda_{i}$ is the rate of arrival of locally generated tasks to $S_{i}$.
- $\mu_{i}>0$ is the average service rate for tasks at the server $S_{i}$. Thus, the average service time per task at $S_{i}$ is $\frac{1}{\mu_{i}}$.
- We define $\rho_{i}=\frac{\lambda_{i}}{\mu_{i}}$, $\lambda =\sum_{i=1}^{n}\lambda_{i}$, and $\mu =\sum_{i=1}^{n}\mu_{i}$.
- $p_{i}$, $0\leq p_{i}\leq 1$, is the probability that, when $S_{i}$ completes the current task that it is executing, it requests to receive a task from the DP.
- $a_{i}$, $0\leq a_{i}\leq 1$, is the probability that the DP accepts $S_{i}$’s request when the DP’s input queue is non-empty.
- $C_{i}=p_{i}{\dot{a}}_{i}$ is the probability that when $S_{i}$ asks for a new task from the DP, it receives it provided that a new task is available at the DP.
- y(t)≥0 is the non-negative integer-valued length of the queue of externally arriving tasks waiting at the Dispatching Platform (DP) at time *t*.
- $y_{i}\left(t\right)\geq 0$ is the integer-valued total number (queue length) of all the tasks that are in the queue at $S_{i}$ at time *t*.
- *k* is a particular value of y(t).
- $k_{i}$ is a particular value of $y_{i}\left(t\right)$, and we define the vectors as follows:
  $$\begin{array}{ccc} & & Y\left(t\right)=(y\left(t\right),y_{1}\left(t\right),\ldots ,y_{n}\left(t\right)),\;\\ & & K=(k,k_{1},\ldots ,k_{n})\;.\; \end{array}$$
- The following vectors are related to $K=(k,k_{1},\ldots ,k_{n})$, *where* $k\geq 0,\;k_{i}\geq 0$:
  (2) $$\begin{array}{ccc} & & K^{-0}=\left(k-1,k_{1},\ldots ,k_{n}\right)\;if\;k>0,\\ & & K^{+0}=\left(k+1,k_{1},\ldots ,k_{n}\right),\\ & & K^{-i}=\left(k,k_{1},\ldots ,k_{i}-1,\ldots ,k_{n}\right)\;if\;k_{i}>0,\\ & & K^{+i}=\left(k,k_{1},\ldots ,k_{i}+1,\ldots ,k_{n}\right)\;. \end{array}$$
- $\Phi_{i}$ is the fraction of external user tasks that the DP allocates to $S_{i}$.
- $\Phi_{i}^{+}$ is the fraction of external user tasks that the DP allocates to $S_{i}$ to minimize the average task response time of the edge system.
- $\Phi_{i}^{*}$ is the fraction of external user tasks that the DP allocates to $S_{i}$ to minimize the average energy consumption per external task assigned to the edge system.
- $X_{i}=\lambda_{i}+\Phi_{i}\Lambda$ is the total arrival rate of tasks to server $S_{i}$, i.e., the load of $S_{i}$.
- $X_{i1}$ is the upper bound for the linear approximation of the power consumption of $S_{i}$, and $X_{i1}<\mu_{i}$
- $q_{i}=\frac{\lambda_{i}+\Phi_{i}\Lambda }{\mu_{i}}$ is the utilization rate of server $S_{i}$. If $q_{i}<0$, it can be interpreted as the probability that $S_{i}$ is busy processing tasks.
- $R_{DP}$ is the average response time at the DP for externally arriving tasks.
- $R_{S}$ is the average response time of all tasks at the *n* servers.
- $\pi_{i0}$ is the power consumption of server $S_{i}$ when the server is idle, i.e., when $X_{i}=0$.
- $\pi_{iM}$ is the maximum power consumption of server $S_{i}$. It is attained when $X_{i}$ is just under the value $\mu_{i}$.
- $\alpha_{i}>0$ is the approximate linear increase in power consumption of $S_{i}$ as a function of the load $X_{i}$.
- $\pi_{i}\left(X_{i}\right)=\pi_{i0}+\alpha_{i}X_{i}$ is the approximate power consumption of $S_{i}$ when its load is $X_{i}$, for $X_{i}<\mu_{i}$.
- $\pi_{i}^{\prime }$ is the derivative of $\pi_{i}\left(X_{i}\right)$ with respect to $\Phi_{i}$.
- $\pi_{i}^{\prime \prime }$ is the second derivative of $\pi_{i}\left(X_{i}\right)$ with respect to $\Phi_{i}$.
- *E* is the average **energy consumption** of the externally arriving tasks that are assigned by the DP to the different servers, and $E=\sum_{i=1}^{n}\Phi_{i}\pi_{i}\left(X_{i}\right)\mu_{i}^{-1}$.

## 3. Analytical Solution for the Dispatching Platform (DP) and Its n Servers

In this section, we construct a G-Network with triggered customer movement [41,42], where the service times at all $S_{i}$ are mutually independent and exponentially distributed random variables, with parameter $\mu_{i}$ for $S_{i}$, and the interarrival times of external tasks to the DP is a Poisson process of rate Λ. The arrivals of local tasks at each $S_{i}$ constitute a mutually independent Poisson process with rate $\lambda_{i}$ and are independent of all the service times at the servers. Thus, in a small time interval of length Δt, an external task arrival occurs to the DP with probability ΛΔt+o(Δt), a local task arrives to any server $S_{i}$ with probability $\lambda_{i}\Delta t+o\left(\Delta t\right)$, and provided that there is a local task at $S_{i}$ (i.e. $k_{i}>0$), a local task ends its service at $S_{i}$ with probability $\mu_{i}\Delta t+o\left(\Delta t\right)$. Here, o(Δt) represents a function that tends to zero with Δt, i.e., $\lim_{\Delta t\to 0}\frac{o(\Delta t)}{\Delta t}=0$.

Also, when a service completes at $S_{i}$, the server requests to receive a new task from the DP with probability $p_{i}$, which is allocated instantaneously with probability $a_{i}$ if k>0 or refused with probability $\left(1-a_{i}\right)$ or accepted with probability $p_{i}$ and not allocated when k=0. Thus, the following state transitions occur:

- $K\to K^{+0}$ with probability ΛΔt+o(Δt).
- $K\to K^{+i}$ with probability $\lambda_{i}\Delta t+o\left(\Delta t\right)$.
- $K^{+0}\to K$ with probability $\mu_{i}C_{i}\Delta t+o\left(\Delta t\right)$ when $k_{i}>0$ (a task at $S_{i}$ departs but is immediately replaced by a task from the DP).
- $K^{+i}\to K$, with probability $\mu_{i}C_{i}\Delta t+o\left(\Delta t\right)$ when k=0 (a task at $S_{i}$ departs; the request for a new task is made but the DP queue is empty (i.e., k=0 and, therefore, the DP has no tasks to send to $S_{i}$).
- $K^{+i}\to K$ with probability $\mu_{i}\left(1-C_{i}\right)\Delta t+o\left(\Delta t\right)$ obtained from
  (3) $$\left[\mu_{i}\left(1-p_{i}\right)+\mu_{i}p_{i}\left(1-a_{i}\right)\right]\Delta t+o\left(\Delta t\right)=\mu_{i}\left(1-C_{i}\right)\Delta t+o\left(\Delta t\right)$$
  independently of the value of *k* or $k_{i}$; note that these values refer to the quantities in the vector $K=(k,k_{1},\ldots ,k_{n})$.
- K→K, with probability $1-\left(\Lambda +\lambda_{i}+\mu_{i}1\left[k_{i}>0\right]\right)\Delta t+o\left(\delta t\right)$.

Then, the probability p(K,t)=Prob[Y(t)=K] satisfies the following system (4) of Chapman–Kolmogorov differential-difference equations:

(4) $$\begin{array}{ccc} \frac{dp(K,t)}{dt} & = & -p\left(K,t\right)[\Lambda +\sum_{i=1}^{n}\left(\mu_{i}1\left[k_{i}>0\right]+\lambda_{i}\right)]+\Lambda p\left(K^{-0},t\right)1\left[k>0\right]\\ & & +\sum_{i=1}^{n}[\lambda_{i}p\left(K^{-i},t\right)1\left[k_{i}>0\right]+\mu_{i}C_{i}p\left(K^{+0},t\right)1\left[k_{i}>0\right]\\ & & +\mu_{i}C_{i}p\left(K^{+i},t\right)1\left[k=0\right]+\mu_{i}\left(1-C_{i}\right)p\left(K^{+i},t\right)]. \end{array}$$

We now state the following result, which we use throughout this paper. **The proof of Theorem 1 is detailed in Appendix A**.

**Theorem 1** (**Key Product Form Result**)**.** *Assume that the arrival processes whose rates are $\Lambda ,\;\lambda_{1},\ldots ,\lambda_{n}$ are all independent Poisson processes and that the service rates $\mu_{i},\;1\leq i\leq n$, are parameters of independent exponentially distributed random variables, which are also independent of the inter-arrival times. Then, if the system of simultaneous non-linear equations*

(5) $$\begin{array}{ccc} & & q=\frac{\Lambda }{\sum_{i=1}^{n}q_{i}\mu_{i}C_{i}},\;\;q_{i}=\frac{\lambda_{i}+qq_{i}\mu_{i}C_{i}}{\mu_{i}}=\frac{\rho_{i}}{1-qC_{i}},\;1\leq i\leq n\;, \end{array}$$

*has a solution that satisfies $0<q<1,\;0<q_{i}<1$,* ***then this solution is unique**, and*

(6) $$\begin{array}{ccc} & & \lim_{t\to \infty }\mathrm{Prob}\left[x\left(t\right)=k,x_{1}\left(t\right)=k_{1},\ldots ,x_{n}\left(t\right)=k_{n}\right]\\ & & \;\;\;\;\;\;\;\;\;\;\;=q^{k}\left(1-q\right)\prod_{i=1}^{n}q_{i}^{k_{i}}\left(1-q_{i}\right), \end{array}$$

*where*

(7) $$q=\lim_{t\to \infty }\mathrm{Prob}\left[x(t)>0\right],\;\;q_{i}=\lim_{t\to \infty }\mathrm{Prob}\left[x_{i}\left(t\right)>0\right].$$

***Note:*** *The denominator of the expression for q in (5) represents the fact that each server $S_{i}$ will notify the DP with probability $p_{i}$ when $S_{i}$’s ongoing job ends, that it is ready to receive a task from the DP, and that the DP will respond by sending a task to $S_{i}$ with probability $a_{i}$ so that $C_{i}=p_{i}\cdot a_{i}$. The rate at which such requests arrive to the DP from $S_{i}$ is, therefore, $q_{i}\mu_{i}p_{i}$, and the rate at which the DP sends tasks to $S_{i}$ is $q_{i}\mu_{i}C_{i}$. Note that both of the equations in (5) are* ***non-linear**, contrary to those of an ordinary “Jackson” (open) or “Gordon–Newell” (closed) product-form queueing network* [43,44]*.*

**Corollary 1.**  *From (6), it is easy to show that when q<1, the average total number of jobs at steady state $N_{DP}$ in the input queue to the DP is*

(8) $$N_{DP}=\frac{q}{1-q}\;,$$

*and the average total number of jobs at steady state $N_{i}$ that are in the input queue of $S_{i}$ is*

(9) $$N_{i}=\frac{q_{i}}{1-q_{i}}.$$

*The expression for $q_{i}$ in (5) has the intuitive property that we now prove; namely, when the stationary solution exists, the total incoming flow of jobs to the DP and the servers $S_{i}$ is identical to the outgoing flow of jobs whose service ends at the n servers, which we use in the proof of Theorem 1 given in Appendix A.*

**Lemma 1.**  *Let us denote*

(10) $$\lambda =\sum_{i=1}^{n}\lambda_{i}\;.$$

*Then, if $0<q_{i}<1,\;0<q<1$, it follows that*

(11) $$\sum_{i=1}^{n}q_{i}\mu_{i}=\Lambda +\lambda \;.$$

**Remark 1.**  *The expression (11) is an intuitive “flow conservation” identity at steady state for a stable system, which states that all the work that arrives at the DP or that arrives locally to the n servers is eventually processed by one of the n servers.*

**Proof of Lemma 1.**  As a consequence of the expressions for *q* and $q_{i}$ in (5), we can write

$$\begin{array}{ccc} \sum_{i=1}^{n}q_{i}\mu_{i} & = & \sum_{i=1}^{n}\lambda_{i}\left[1+\sum_{l=1}^{\infty }{\left(qC_{i}\right)}^{l}\right], \end{array}$$

and using the expression for *q* in (5), we obtain

(12) $$\begin{array}{ccc} \sum_{i=1}^{n}q_{i}\mu_{i} & = & \lambda +\frac{\Lambda }{\sum_{j=1}^{n}q_{j}\mu_{j}C_{j}}.\sum_{i=1}^{n}\frac{\lambda_{i}}{1-qC_{i}}=\lambda +\frac{\Lambda }{\sum_{j=1}^{n}q_{j}\mu_{j}C_{j}}.\sum_{j=1}^{n}q_{j}\mu_{j}C_{j}=\lambda +\Lambda , \end{array}$$

which completes the proof. □

**Corollary of Lemma 1.** *Since we assume that* $0<q_{i}<1,\;1\leq i\leq n$, the following holds:

(13) $$\begin{array}{ccc} & & Denoting\;\rho_{i}=\frac{\lambda_{i}}{\mu_{i}},\;we\;have:\rho_{i}<1-qC_{i},\;and\;hence\;C_{i}<\frac{1-\rho_{i}}{q}. \end{array}$$

## 4. Minimizing the Average Response Time or Average Delay at the DP

The well-known “Little’s Formula” [45] can be used to compute the average response time of tasks entering through the DP and of tasks entering the edge system composed of *n* servers. Here, Λ is the total arrival rate of externally arriving tasks to the DP and $qq_{i}\mu_{i}C_{i}$ is the arrival rate of tasks from the DP to server $S_{i}$.

Since Λ is the total arrival rate of such tasks, if $R_{DP}$ denotes the average response time of tasks at the DP before they are assigned to a server, by Little’s Formula and Equation (8) in Corollary 1, we have

(14) $$\begin{array}{ccc} R_{DP} & = & \frac{N_{DP}}{\Lambda }=\frac{1}{\Lambda }\frac{q}{1-q},\; \end{array}$$

and we would like to know how we should choose $C_{i},\;i=1,\ldots ,n$, to minimize $R_{DP}$. To this effect, the following result is needed:

**Theorem 2.**  *Let $0<q_{i}<1$, and denote $D_{i}=\frac{dq}{dC_{i}},\;d_{ij}=\frac{dD_{i}}{dC_{j}}$. It follows that $D_{i}<0$, $d_{ij}<0$, and $d_{ii}>0$ for i,j=1,…n,j≠i.*

***The proof of Theorem 2 is given in Appendix B.** *

*Using (14), we can derive*

(15) $$\frac{dR_{DP}}{dC_{i}}=\frac{1}{\Lambda }\frac{D_{i}}{1-q},\;\frac{d^{2}R_{DP}}{dC_{i}^{2}}=\frac{1}{\Lambda }\frac{d_{ii}\left(1-q\right)+D_{i}^{2}}{{\left(1-q\right)}^{2}}.$$

*Then, also using Theorem 2, we have $\frac{dR_{DP}}{dC_{i}}<0$ and $\frac{d^{2}R_{DP}}{dC_{i}^{2}}>0$ for i=1,…,n.*

**Theorem 3.**  *Using (14), (15), and Theorem 2, it follows that for fixed *Λ*, the average response time $R_{DP}$ for a task that arrives from the MBS or an external user to the DP, until it is assigned to one of the server input queues, is minimized with respect to $0\leq C_{i}\leq 1$ by taking the largest possible value of $C_{i}$, which is $C_{i}=1$. When all the $C_{i},\;1\leq i\leq n$, are set to $C_{i}=1$, then $R_{DP}$ attains its minimum value wth respect to the vector $C=(C_{1},\ldots ,C_{n})$.*

## 5. Minimizing the Average Response Time $R_{S}$ at the Edge Servers

Different edge servers have different task processing rates $\mu_{i}$ and different local task arrival rates $\lambda_{i}$. Therefore, it is worth understanding how the DP should share the tasks that it receives among the edge servers to achieve a minimum average response time $R_{S}$ for **all the tasks**, both those that arrive locally to each server and those that are assigned by the DP. Let $\Phi_{i}$ denote the proportion of incoming external tasks that the DP assigns to server $S_{i}$:

(16) $$\Phi_{i}=\frac{q_{i}\mu_{i}C_{i}}{\sum_{j=1}^{n}q_{j}\mu_{j}C_{j}},\;\;\sum_{j=1}^{n}\Phi_{j}=1,$$

so that the total arrival rate of tasks arriving to reach $S_{i}$ is $\lambda_{i}+\Lambda \Phi_{i}$. As a result, when q<1, $q_{i}<1,\;i=1,\ldots ,n$, in steady state, the average number of tasks $N_{S}$ at the *n* servers can be obtained from (6) in Theorem 1 as

(17) $$N_{S}=\sum_{i=1}^{n}N_{i}=\sum_{i=1}^{n}\frac{q_{i}}{1-q_{i}},\;where\;q_{i}=\frac{\lambda_{i}+\Lambda \Phi_{i}}{\mu_{i}}\;,$$

and by Little’s Theorem, we have

(18) $$R_{S}=\frac{1}{\Lambda +\lambda }\sum_{i=1}^{n}\frac{q_{i}}{1-q_{i}}=\frac{1}{\Lambda +\lambda }\sum_{i=1}^{n}\frac{\lambda_{i}+\Lambda \Phi_{i}}{\mu_{i}-\lambda_{i}-\Lambda \Phi_{i}},\;where\;\lambda =\sum_{i=1}^{n}\lambda_{i}\;.$$

We can now state the following result, **whose proof is given in Appendix C**.

**Theorem 4.**  *Let $0\leq q<1,\;0\leq q_{j}<1$ for 1≤j≤n. Then, the average response time at steady state for all tasks that are processed by the n servers, denoted by $R_{S}$,* ***attains its global minimum with respect to the vector** ***$\Phi =(\Phi_{1},\ldots ,\Phi_{n})$** ***when** ***$\Phi_{j}$** ***is equal to*** $\Phi_{j}^{*}$ *:*

(19) $$\begin{array}{ccc} \Phi_{j}^{*} & = & \frac{\mu_{j}-\lambda_{j}}{\Lambda }-\frac{\mu -\Lambda -\lambda }{\Lambda }\frac{\sqrt{\frac{\mu_{j}}{\mu_{1}}}}{\left[\sum_{i=1}^{n}\sqrt{\frac{\mu_{i}}{\mu_{1}}}\right]},\;1\leq j\leq n,\;where\;\mu =\sum_{j=1}^{n}\mu_{j}\;,\\ & = & \frac{\sqrt{\frac{\mu_{j}}{\mu_{1}}}}{\left[\sum_{i=1}^{n}\sqrt{\frac{\mu_{i}}{\mu_{1}}}\right]}+\frac{1}{\Lambda }[\mu_{j}-\lambda_{j}-\left(\mu -\lambda \right)\frac{\sqrt{\frac{\mu_{j}}{\mu_{1}}}}{\left[\sum_{i=1}^{n}\sqrt{\frac{\mu_{i}}{\mu_{1}}}\right]}]\;,\;1\leq j\leq n\;.\; \end{array}$$

***Communication Overhead and Computational Cost.*** *From (19), we see that the terms*

(20) $$\mu \;and\;\frac{\sqrt{\frac{\mu_{j}}{\mu_{1}}}}{\left[\sum_{i=1}^{n}\sqrt{\frac{\mu_{i}}{\mu_{1}}}\right]},$$

can be computed in advance once and for all for a given set of n servers since they only depend on the server speed parameters $\mu_{i},\;i=1,\ldots ,n$, and do not need to be re-computed for each decision. *Λ* is known by the DP, which locally monitors the external arrival rate of tasks, and no communication is needed to update *Λ*. The parameters $\lambda_{j}$ must be updated in (19) and should be sent by each $S_{j}$ to the DP (where the task assignment decision is taken) each time $\lambda_{j}$ changes. This boils down to a periodic communication overhead of, at most, a total of n packets that are sent from the servers to the DP. From a computational standpoint, obtaining (19) only requires four additions and subtractions and two multiplications for each of the n values $\Phi_{j}^{*}$.

**Corollary 2.**  The minimum value of $R_{S}$, denoted $R_{S}^{*}$ is

(21) $$\begin{array}{c} R_{S}^{*}=\frac{1}{\Lambda +\lambda }\sum_{j=1}^{n}\frac{\lambda_{j}+\Lambda \Phi_{j}^{*}}{\mu_{j}-\lambda_{j}-\Lambda \Phi_{j}^{*}}=\frac{1}{\Lambda +\lambda }\sum_{j=1}^{n}\frac{\mu_{j}}{\mu_{j}-\sqrt{\frac{\mu_{j}}{\mu_{1}}}\lambda_{j}-\Lambda \Phi_{j}^{*}}. \end{array}$$

**Corollary 3.**  *In many cases of interest, an edge system is composed **of the DP and** **n** **identical servers** ***$S_{i}$,** ***which, in general, have different local loads*** **$\lambda_{i}$**  *so that we have $\mu_{i}=\mu ,\;1\leq i\leq n$. In this case, $R_{S}$ is minimized when*

(22) $$\Phi_{i}^{*}=\Phi_{1}^{*}+\frac{\lambda_{1}-\lambda_{i}}{\Lambda },\;2\leq i\leq n,\;\;\Phi_{1}^{*}=\frac{1}{n}\left[1+\frac{\sum_{i=2}^{n}\left(\lambda_{i}-\lambda_{1}\right)}{\Lambda }\right]\;.$$

## 6. Minimizing Energy Consumption

**Figure 2:**
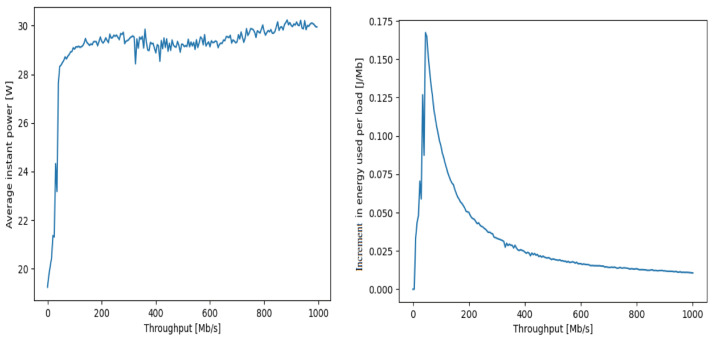
The curve on the left shows the power consumption that was measured on an NUC versus its overall arrival rate of workload. There is a substantial power consumption of close to 63% of its maximum value when the NUC is idle. We observe that the power consumption attains its maximum value of 30 Watts as the workload increases. The curve on the right shows the corresponding energy consumption per arriving request in Joules as a function of the load.

An important system performance metric of interest is the energy consumption of the system. As an example, the measured power and energy consumption characteristics of an Intel NUC processor [46] that is widely used in edge systems are shown in Figure 2 based on accurate measurements that were reported in [47].

Let us note from (11) and (12) that Λ is the total arrival rate of external tasks to the DP; these are, in turn, assigned by the DP to the *n* edge servers. Also, we define $X_{i}=\lambda_{i}+\Lambda \Phi_{i}$, where (as previously in this paper) $\lambda_{i}$ is the local arrival rate of tasks to $S_{i}$ and $\Phi_{i}$ is the fraction of externally arriving tasks that are allocated by the DP to $S_{i}$.

The curve on the left in Figure 2 shows the rise in the power consumption as a function of its load, expressed as the arrival rate of workload to the NUC, starting from a value of roughly 19 Watts when the NUC is idle and attaining a maximum value of approximately 30 Watts when the NUC is fully loaded. The curve on the right in Figure 2 shows the energy consumption in Joules per arriving request as a function of the total arrival rate of tasks $X_{i}$ to server $S_{i}$.

**Figure 3:**
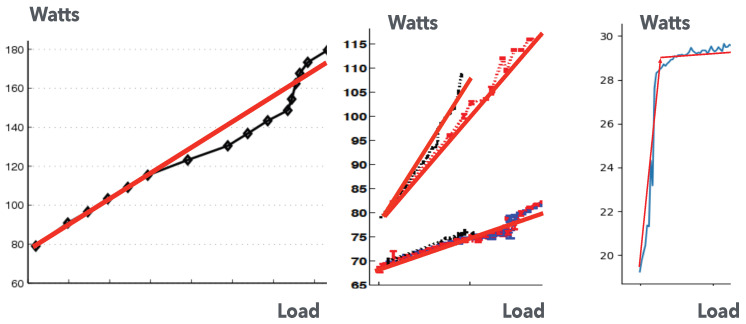
We illustrate the measured characteristics of the power consumption $\Pi_{i}\left(X_{i}\right)$ along the y-axis in Watts, versus the load $X_{i}$ along the x-axis in tasks/sec for several different servers, showing the approximately linear increase in power consumption at some rate $\alpha_{i}>0$, which depends on the characteristics of the different processors, between the zero load level (no task arrivals and the server is idle), which corresponds to $\pi_{i0}$, up to close to the maximum value of the power consumption that we denote by $\pi_{iM}$. Note that the value $X_{1i}$ cannot exceed the maximum processing rate of jobs $\mu_{i}$ of $S_{i}$. The linear characteristic is displayed as a straight red line on top of the measured data that are also shown in the figure. The rightmost curve refers to the NUC whose characteristics are discussed in Figure 2.

Indeed, the curve on the left-hand-side of Figure 2 and the different measurement curves shown in Figure 3 also suggest the following representation for the power consumption $\pi_{i}\left(X_{i}\right)$ of server $S_{i}$ (23), where $X_{i}=\lambda_{i}+\Lambda \Phi_{i}$, rising from the power consumption $\pi_{i0}$ when $S_{i}$ is idle, up to its maximum power consumption denoted by $\pi_{iM}$. Thus, these measurement results indicate that the power versus workload characteristics of a server may be represented by a piece-wise linear approximation consisting of a straight line from $X_{i}=0$ to $X_{i}=X_{i1}$ with a positive slope and a second flat (nearly zero slope) straight line from $X_{i1}$ to higher values of $X_{i}$. Also, $X_{i1}$ is smaller than the maximum processing or service rate $\mu_{i}$ of server *i*. We, therefore, use this observation to express the approximation for $0\leq X_{i}\leq X_{i1}$ with $\pi_{i}\left(X_{i1}\right)=\pi_{iM}$ as

(23) $$\begin{array}{ccc} \pi_{i}\left(X_{i}\right) & = & \pi_{i0},\;if\;\;X_{i}=0,\\ & = & \pi_{i0}+\alpha_{i}X_{i}\;,\;if\;\;0\leq X_{i}\leq X_{i1}<\mu_{i}\;, \end{array}$$

where $\alpha_{i}>0$ is a positive constant that depends on the specific server being considered. We can then define the first and second derivatives of $\pi_{i}\left(X_{i}\right)$ with respect to $\Phi_{i}$:

(24) $$\pi_{i}^{\prime }=\frac{d\pi_{i}\left(X_{i}\right)}{d\Phi_{i}},\;\pi_{i}^{\prime \prime }=\frac{d^{2}\pi_{i}\left(X_{i}\right)}{d\Phi_{i}^{2}}\;.$$

When i≠1, we have, for $X_{i}<\mu_{i}$,

(25) $$\pi_{i}^{\prime }=\alpha_{i}\Lambda ,\;\pi_{i}^{\prime \prime }=0,\;\;for\;\;\alpha_{i}>0,\;when\;0\leq X_{i}<X_{i1}\;.$$

Also, since $\Phi_{1}=1-\sum_{i=2}^{n}\Phi_{i}$, we have $\frac{d\Phi_{1}}{d\Phi_{i}}=-1$ for i≠1. Thus, the first and second derivatives of $\pi_{1}\left(X_{1}\right)$ with respect to $\Phi_{i}$ for i≠1 are

(26) $$\frac{d\pi_{1}\left(X_{1}\right)}{d\Phi_{i}}=-\alpha_{1}\Lambda ,\;for\;\alpha_{1}>0,\;\frac{d^{2}\pi_{1}\left(X_{1}\right)}{d\Phi_{i}^{2}}=0,\;for\;0\leq X_{1}<X_{11}.$$

### Allocating Incoming Tasks to Minimize the Average Additional Energy Consumed by the Servers

If the DP sends an externally arriving task to server $S_{i}$, we know that the task waits for some time, and then it will be processed during $\mu_{i}^{-1}$ time units on average. If the power consumption of $S_{i}$ is $\pi_{i}$ and $\Phi_{i}$ is the probability that the DP has chosen to send the task to $S_{i}$, then the energy that is consumed by the task is simply $\pi_{i}\times \mu_{i}^{-1}$.

Therefore, the expected average energy consumption *E* for executing a task sent from the DP to the edge system composed of *n* servers is

(27) $$E=\sum_{i=1}^{n}\left[\;\Phi_{i}\times \frac{\pi_{i}\left(X_{i}\right)}{\mu_{i}}\right].$$

This leads us directly to the following result, **whose proof is given in Appendix D.**

**Theorem 5.**  *Assuming the power consumption characteristic given in (23), the proportion of incoming traffic that should be allocated to server $S_{i}$ to**minimize*** *E for j=2,…,n is*

(28) $$\Phi_{j}^{+}=\Phi_{1}^{+}\frac{\alpha_{1}\mu_{j}}{\alpha_{j}\mu_{1}}+\frac{1}{2\Lambda \alpha_{j}}\;\left[\pi_{10}\frac{\mu_{j}}{\mu_{1}}-\pi_{j0}\right]\;,$$

*where*

(29) $$\Phi_{1}^{+}=\frac{1+\frac{1}{2\Lambda }\sum_{i=2}^{n}\left[\frac{\pi_{i0}}{\alpha_{i}}-\frac{\pi_{10}}{\mu_{1}}\frac{\mu_{i}}{\alpha_{i}}\right]}{1+\frac{\alpha_{1}}{\mu_{1}}\sum_{i=2}^{n}\frac{\mu_{i}}{\alpha_{i}}}\;.$$

*As would be expected, when all the servers are identical with $\pi_{i0}=\pi_{i1},\;\alpha_{i}=\alpha_{1},\;\mu_{i}=\mu_{1}$ for i=2,…,n, we have $\Phi_{1}^{+}=\frac{1}{n}$, and $\Phi_{j}^{+}=\Phi_{1}^{+},\;2\leq j\leq n$.*

***Communication Overhead and Computational Overhead.***  *Since the parameters $\alpha_{j},\;\mu_{j},\;\pi_{i0}$ are fixed and can be known in advance for the servers $S_{j},\;j=1,\ldots ,n$, the terms $\sum_{i=2}^{n}\left[\frac{\pi_{i0}}{\alpha_{i}}-\frac{\pi_{10}}{\mu_{1}}\frac{\mu_{i}}{\alpha_{i}}\right]$, $1+\frac{\alpha_{1}}{\mu_{1}}\sum_{i=2}^{n}\frac{\mu_{i}}{\alpha_{i}}$, $\frac{\alpha_{1}\mu_{j}}{\alpha_{j}\mu_{1}}$, and $\frac{1}{2\alpha_{j}}\;\left[\pi_{10}\frac{\mu_{j}}{\mu_{1}}-\pi_{j0}\right]$ can be computed just one time in advance for j=2,…,n. The only parameter in (28) and (29) that must be measured is *Λ*; it is measured directly by the DP, which uses it to compute the values of $\Phi_{j}$ that minimize E. Therefore, there is no communication overhead involved in choosing the fraction of externally arriving tasks assigned to each server to minimize the additional average energy consumption E. Considering the computational overhead, we note that the computation of $\Phi_{i}^{+}$ involves an additional addition and two divisions. The computation of each of the remaining $\Phi_{j}^{+}$ involves one additional multiplication, one division, and one addition. Thus, we see that the number of arithmetic operations needed to compute all of the n values of $\Phi_{j}^{+}$ is 3n for each new value of *Λ*.*

## 7. Conclusions

Edge computing systems, composed of clusters of processors, are particularly important for supporting the low latency, high throughput, and low power consumption needs of mobile base stations and other communication systems. Their aim is to provide crucial low latency and sustainable low energy consuming services for the Internet of Things and support the transition of communications to 5G and 6th generation (6G) mobile networks. Thus, considerable work has been devoted to the design of different types of algorithms for configuring them, dynamically or statically, to optimize the allocation of tasks to edge system servers.

Much prior work has used machine learning, including reinforcement learning, non-linear optimization methods, and market-based mechanisms, and some of these methods have been tested in experimental environments. Though this work has been extremely useful in generating experience about the manner in which edge systems can be implemented, it comes at the cost of extensive simulations and time-consuming real-system experimentations. Furthermore, the machine learning-based approaches, such as that in our earlier work [10,47], do not provide insight into the fraction of tasks that should be allocated to different servers to achieve optimality.

Thus, in the present work, we address the edge computing design process through an analytical model that results in explicit formulas for optimal task allocation, minimal task latency, and minimal energy consumption of the system as a whole. We show that this approach leads to simple formulas that provide the optimum share of externally arriving tasks that should be assigned to each edge server. We also observe that these formulas are computationally very simple and that they lead to very low communication overhead. In future work, we plan to prioritize the execution of locally generated tasks and remote tasks and include the effect of different types of tasks being executed in the system.

We also plan to implement the proposed algorithms in an experimental test bed and compare various machine learning-based algorithms and other simple heuristics (such as greedy algorithms) to see how close they can get to achieving the optimum performance obtained via the analytical approach.

## Data Availability

The data presented in this study are available on request from the author.

## Appendix A

**Proof of Theorem 1 (Key Product Form Result).** For the Equation (4) at steady state, we set $\frac{dp(K,t)}{dt}=0$ and drop the dependency on *t* to write

(A1) $$\begin{array}{ccc} & & p\left(K\right)[\Lambda +\sum_{i=1}^{n}\left(\mu_{i}1\left[k_{i}>0\right]+\lambda_{i}\right)]\\ & & =\Lambda p\left(K^{-0}\right)1\left[k>0\right]+\sum_{i=1}^{n}[\lambda_{i}p\left(K^{-i}\right)1\left[k_{i}>0\right]\\ & & +\mu_{i}C_{i}p\left(K^{+0}\right)1\left[k_{i}>0\right]+\mu_{i}C_{i}p\left(K^{+i}\right)1\left[k=0\right]\\ & & +\mu_{i}\left(1-C_{i}\right)p\left(K^{+i}\right)].\; \end{array}$$

Then, we divide both sides of (A1) by p(K) and substitute the expression from (6), to obtain

$$\begin{array}{ccc} & & \left[\Lambda +\sum_{i=1}^{n}\left(\mu_{i}1\left[k_{i}>0\right]+\lambda_{i}\right)\right]=\frac{\Lambda }{q}1\left[k>0\right]\\ & & +\sum_{i=1}^{n}[\frac{\lambda_{i}}{q_{i}}1\left[k_{i}>0\right]+\mu_{i}C_{i}q1\left[k_{i}>0\right]+\mu_{i}C_{i}q_{i}1\left[k=0\right]\\ & & +\mu_{i}\left(1-C_{i}\right)q_{i}]. \end{array}$$

Now, substituting $\mu_{i}q_{i}=\frac{\lambda_{i}}{1-qC_{i}}$ from the expression for $q_{i}$ in (5) and the expression $q=\Lambda \sum_{i=1}^{n}q_{i}\mu_{i}C_{i}$, we have

$$\begin{array}{ccc} & & [\Lambda +\sum_{i=1}^{n}\left(\mu_{i}1\left[k_{i}>0\right]+\lambda_{i}\right)]=\sum_{i=1}^{n}q_{i}\mu_{i}C_{i}1\left[k>0\right]\\ & & +\sum_{i=1}^{n}[\mu_{i}\left(1-qC_{i}\right)1\left[k_{i}>0\right]+\mu_{i}C_{i}q1\left[k_{i}>0\right]+\mu_{i}C_{i}q_{i}1\left[k=0\right]\\ & & +\mu_{i}\left(1-C_{i}\right)q_{i}], \end{array}$$

or canceling identical terms with opposite signs and summing identical terms for k>0 and k=0], we obtain

$$\begin{array}{ccc} & & [\Lambda +\sum_{i=1}^{n}\left(\mu_{i}1\left[k_{i}>0\right]+\lambda_{i}\right)]=\sum_{i=1}^{n}q_{i}\mu_{i}C_{i}1\\ & & +\sum_{i=1}^{n}\left[\mu_{i}1\left[k_{i}>0\right]+\mu_{i}\left(1-C_{i}\right)q_{i}\right]. \end{array}$$

Now, canceling identical terms on both sides of the equation and also canceling identical terms with opposite signs on the right-hand side, we are left with

$$\begin{array}{c} \Lambda +\sum_{i=1}^{n}\lambda_{i}=\sum_{i=1}^{n}\mu_{i}q_{i}. \end{array}$$

However, by **Lemma 1**, the right-hand side and left-hand side of the above equation are identical; hence, the solution (5) and (6) has now been proved. The **uniqueness** of the solutions of the non-linear Equation (5) follows from the known uniqueness of the stationary solution of the Chapman–Kolmogorov differential-difference Equation (4) [48,49]. This completes the **proof of the Key Product Form (Theorem 1).** □

## Appendix B

**Proof of Theorem 2.** We use (5) to derive

(A2) $$\begin{array}{ccc} D_{i} & = & -\frac{\Lambda [\sum_{j=1}^{n}d_{ji}\mu_{j}C_{j}+q_{i}\mu_{i}]}{{\left[\sum_{j=1}^{n}q_{j}\mu_{j}C_{j}\right]}^{2}},\\ & = & -\frac{q^{2}}{\Lambda }\left[\sum_{j=1}^{n}d_{ji}\mu_{j}C_{j}+q_{i}\mu_{i}\right], \end{array}$$

(A3) $$\begin{array}{ccc} d_{ji} & = & \rho_{j}\frac{D_{i}C_{j}+q1\left[i=j\right]}{{\left[1-qC_{j}\right]}^{2}},\\ & = & \frac{q_{j}^{2}}{\rho_{j}}\left[D_{i}C_{j}+q1\left[i=j\right]\right]. \end{array}$$

As a consequence, we can write

(A4) $$\begin{array}{ccc} D_{i} & = & -\frac{q^{2}}{\Lambda }\left[\sum_{j=1}^{n}\frac{q_{j}^{2}}{\rho_{j}}D_{i}\mu_{j}C_{j}^{2}+\frac{q_{i}^{2}}{\rho_{i}}q\mu_{i}C_{i}+q_{i}\mu_{i}\right],\\ & = & -q^{2}\frac{q_{i}\mu_{i}\left[1+\frac{q_{i}}{\rho_{i}}qC_{i}\right]}{\Lambda +q^{2}\sum_{j=1}^{n}\frac{q_{j}^{2}}{\rho_{j}}\mu_{j}C_{j}^{2}}\\ & = & -q\frac{q_{i}\mu_{i}\left[1+\frac{q_{i}}{\rho_{i}}qC_{i}\right]}{\sum_{j=1}^{n}q_{j}\mu_{j}C_{j}\left[1+q\frac{q_{j}}{\rho_{j}}C_{j}\right]},\\ & = & -q\frac{\frac{\lambda_{i}}{{\left(1-qC_{i}\right)}^{2}}}{\sum_{j=1}^{n}\frac{\lambda_{j}C_{j}}{{\left(1-qC_{j}\right)}^{2}}}. \end{array}$$

**Thus, (A4) tells us that if q>0 and all the $q_{i}>0$, then all $D_{i}<0$.**
Now, substituting (A4) back into (A3), we have

(A5) $$\begin{array}{ccc} d_{ji} & = & q[\frac{q_{j}^{2}}{\rho_{j}}1\left[i=j\right]-\frac{q_{j}^{2}}{\rho_{j}}\frac{\frac{\lambda_{i}C_{j}}{{\left(1-qC_{i}\right)}^{2}}}{\sum_{l=1}^{n}\frac{\lambda_{l}C_{l}}{{\left(1-qC_{l}\right)}^{2}}}],\\ & = & q[\frac{q_{j}^{2}}{\rho_{j}}1\left[i=j\right]-\frac{q_{i}^{2}\mu_{i}}{\rho_{i}}\frac{\frac{q_{j}^{2}C_{j}}{\rho_{j}}}{\sum_{l=1}^{n}\frac{q_{l}^{2}\mu_{l}C_{l}}{\rho_{l}}}],\\ & = & q\frac{q_{i}^{2}}{\rho_{i}}\left[1\left[i=j\right]-\mu_{i}\frac{\frac{q_{j}^{2}C_{j}}{\rho_{j}}}{\sum_{l=1}^{n}\frac{q_{l}^{2}\mu_{l}C_{l}}{\rho_{l}}}\right]. \end{array}$$

**Since the first term (which is non-negative) in (A5) vanishes when i≠j, we can see that $d_{ji}<0$ for i≠j.** The last part of the proof must establish that $d_{ii}>0$. Using (A5), we write

$$\begin{array}{ccc} d_{ii} & = & q\frac{q_{i}^{2}}{\rho_{i}}\left[1-\frac{\frac{q_{i}^{2}\mu_{i}C_{i}}{\rho_{j}}}{\sum_{l=1}^{n}\frac{q_{l}^{2}\mu_{l}C_{l}}{\rho_{l}}}\right], \end{array}$$

so that $d_{ii}>0$ is obvious as long as n>1, $0<q_{l}<1$ and all $C_{l}>0$. **Hence, under these conditions, we have $d_{ii}>0$**. This completes the proof of Theorem 2. □

## Appendix C

**Proof of Theorem 3.** We start from (16) and (18) to write

(A6) $$\begin{array}{ccc} R_{S} & = & \frac{1}{\Lambda +\lambda }\sum_{i=1}^{n}\frac{\lambda_{j}+\Lambda \Phi_{i}}{\mu_{i}-\lambda_{i}-\Lambda \Phi_{i}},\;with\;\Phi_{1}=1-\sum_{i=2}^{n}\Phi_{i}\;, \end{array}$$

so that for 2≤i≤n we have $\frac{d\Phi_{1}}{d\Phi_{i}}=-1$ and

(A7) $$\begin{array}{ccc} \frac{dR_{S}}{d\Phi_{i}} & = & \frac{\Lambda \left(\mu_{i}-\lambda_{i}-\Lambda \Phi_{i}\right)+\Lambda \left(\lambda_{i}+\Lambda \Phi_{i}\right)}{{\left(\mu_{i}-\lambda_{i}-\Lambda \Phi_{i}\right)}^{2}}\\ & & \frac{1}{\Lambda +\lambda }\left[\frac{\Lambda \left(\mu_{1}-\lambda_{1}-\Lambda \Phi_{1}\right)+\Lambda \left(\lambda_{1}+\Lambda \Phi_{1}\right)}{{\left(\mu_{1}-\lambda_{1}-\Lambda \Phi_{1}\right)}^{2}}\right]\;,\\ & = & \frac{\Lambda }{\Lambda +\lambda }\left[\frac{\mu_{i}}{{\left(\mu_{i}-\lambda_{i}-\Lambda \Phi_{i}\right)}^{2}}-\frac{\mu_{1}}{{\left(\mu_{1}-\lambda_{1}-\Lambda \Phi_{1}\right)}^{2}}\right]\;, \end{array}$$

(A8) $$\begin{array}{ccc} \frac{d^{2}R_{S}}{d\Phi_{i}^{2}} & = & \frac{\Lambda^{2}}{\Lambda +\lambda }\left[\frac{\mu_{i}}{{\left(\mu_{i}-\lambda_{i}-\Lambda \Phi_{i}\right)}^{3}}+\frac{\mu_{1}}{{\left(\mu_{1}-\lambda_{1}-\Lambda \Phi_{1}\right)}^{3}}\right]. \end{array}$$

Since $q_{i}<1$ for all 1≤i≤n, it follows from (A8) that $\frac{d^{2}R_{S}}{d\Phi_{i}^{2}}>0$. Therefore, the minimum of $R_{S}$ with respect to $\Phi_{i}\;,\;i=1,\ldots ,n$, is obtained from (A7) when

(A9) $$\begin{array}{ccc} & & \frac{dR_{S}}{d\Phi_{i}}=0,\;or\;\;\left(\mu_{1}-\lambda_{1}-\Lambda \Phi_{1}^{*}\right)\sqrt{\frac{\mu_{i}}{\mu_{1}}}=\mu_{i}-\lambda_{i}-\Lambda \Phi_{i}^{*}\;. \end{array}$$

Using $\Phi_{1}^{*}=1-\sum_{i=2}^{n}\Phi_{i}^{*}$ and summing both sides of (A9) over 2≤i≤n, we have

(A10) $$\begin{array}{ccc} & & \left(\mu_{1}-\lambda_{1}-\Lambda \Phi_{1}^{*}\right)\sum_{i=2}^{n}\sqrt{\frac{\mu_{i}}{\mu_{1}}}=\mu -\mu_{1}-\lambda +\lambda_{1}-\Lambda +\Lambda \Phi_{1}^{*}\;,\;\;or\\ & & \Lambda \Phi_{1}^{*}\left[1+\sum_{i=2}^{n}\sqrt{\frac{\mu_{i}}{\mu_{1}}}\right]=\left(\mu_{1}-\lambda_{1}\right)\left[1+\sum_{i=2}^{n}\sqrt{\frac{\mu_{i}}{\mu_{1}}}\right]+\left(\mu -\Lambda -\lambda \right),\;or\\ \Lambda \Phi_{1}^{*} & = & \mu_{1}-\lambda_{1}-\frac{\mu -\Lambda -\lambda }{1+\sum_{i=2}^{n}\sqrt{\frac{\mu_{i}}{\mu_{1}}}},\;and\;\Phi_{i}^{*}=\frac{\mu_{i}-\lambda_{i}}{\Lambda }-\frac{\mu -\Lambda -\lambda }{\Lambda }\frac{\sqrt{\frac{\mu_{i}}{\mu_{1}}}}{\sum_{j=1}^{n}\sqrt{\frac{\mu_{j}}{\mu_{1}}}}\;, \end{array}$$

and the proof is complete. □

## Appendix D

**Proof of Theorem 4.** Let us use the notation $E_{i}^{\prime },\;E_{i}^{\prime \prime },\;and\;\pi_{i}^{\prime }\;to\;denote\;\frac{dE}{d\Phi_{i}},\;\frac{d^{2}E}{d\Phi_{i}^{2}},\;and\;\frac{d\pi_{i}}{d\Phi_{i}}$, respectively, 1≤j≤n. Using the fact that $\sum_{j=1}^{n}\Phi_{j}=1$, we obtain the following expressions for i≠1:

(A11) $$\begin{array}{ccc} & & E_{i}^{\prime }=\frac{\pi_{i}}{\mu_{i}}+\Phi_{i}\times \frac{\pi_{i}^{\prime }}{\mu_{i}}-\frac{\pi_{1}}{\mu_{1}}-\Phi_{1}\times \frac{\pi_{1}^{\prime }}{\mu_{1}}, \end{array}$$

(A12) $$\begin{array}{ccc} & & E_{i}^{\prime \prime }=\frac{\pi_{i}^{\prime }}{\mu_{i}}+\Phi_{i}\times \frac{\pi_{i}^{\prime \prime }}{\mu_{i}}+\frac{\pi_{i}^{\prime }}{\mu_{i}}+\frac{\pi_{1}^{\prime }}{\mu_{1}}+\frac{\pi_{i}^{\prime }}{\mu_{i}}+\Phi_{1}\times \frac{\pi_{1}^{\prime \prime }}{\mu_{1}}\;. \end{array}$$

We see easily that $E_{i}^{\prime \prime }>0$ when $0\leq X_{i}<X_{i1}$ for i≠1. Thus, for i≠1, the value $\Phi_{i}^{+}$ of $\Phi_{i}$ that minimizes *E* is attained by setting $E_{i}^{\prime }=0$ in (A11), leading to

(A13) $$\begin{array}{ccc} \Phi_{i}^{+}\frac{\pi_{i}^{\prime }}{\mu_{i}} & = & \Phi_{1}^{+}\frac{\pi_{1}^{\prime }}{\mu_{1}}+\frac{\pi_{1}}{\mu_{1}}-\frac{\pi_{i}}{\mu_{i}}\;,\;or\;\\ \Phi_{i}^{+} & = & \Phi_{1}\frac{\alpha_{1}\mu_{i}}{\alpha_{i}\mu_{1}}+\mu_{i}\frac{\pi_{10}+\alpha_{1}\lambda_{1}+\alpha_{1}\Phi_{1}^{+}\Lambda }{\alpha_{i}\Lambda \mu_{1}}-\frac{\pi_{i0}+\alpha_{i}\lambda_{i}+\alpha_{i}\Phi_{i}^{+}\Lambda }{\alpha_{i}\Lambda },\\ 2\Phi_{i}^{+} & = & 2\Phi_{1}^{+}\frac{\mu_{i}\alpha_{1}}{\mu_{1}\alpha_{i}}+\frac{\lambda_{1}\mu_{i}\alpha_{1}}{\Lambda \mu_{1}\alpha_{i}}-\frac{\lambda_{i}}{\Lambda }+\frac{\frac{\mu_{i}}{\mu_{1}}\pi_{10}-\pi_{i0}}{\alpha_{i}\Lambda }\;,\;yielding\\ \Phi_{i}^{+} & = & \Phi_{1}^{+}\frac{\mu_{i}\alpha_{1}}{\mu_{1}\alpha_{i}}+\frac{\frac{\mu_{i}}{\mu_{1}}\pi_{10}-\pi_{i0}}{\alpha_{i}\Lambda }\;. \end{array}$$

Summing both sides of (A13) from 2 to *n*, we obtain

$$\begin{array}{ccc} 1-\Phi_{1}^{+} & = & \Phi_{1}^{+}\frac{\alpha_{1}}{\mu_{1}}\sum_{2}^{n}\frac{\mu_{i}}{\alpha_{i}}+\frac{\pi_{1}}{\Lambda \mu_{1}}\sum_{2}^{n}\frac{\mu_{i}}{\alpha_{i}}-\sum_{2}^{n}\frac{\pi_{i}}{\Lambda \alpha_{i}}\\ & = & \Phi_{1}^{+}\frac{\alpha_{1}}{\mu_{1}}\sum_{2}^{n}\frac{\mu_{i}}{\alpha_{i}}+\frac{\pi_{1}}{\Lambda \mu_{1}}\sum_{2}^{n}\frac{\mu_{i}}{\alpha_{i}}-\sum_{2}^{n}\frac{\pi_{i0}}{\Lambda \alpha_{i}}-\sum_{2}^{n}\Phi_{i}^{+},\;implying\;that:\\ 2(1-\Phi_{1}^{+}) & = & \Phi_{1}^{+}\frac{\alpha_{1}}{\mu_{1}}\sum_{2}^{n}\frac{\mu_{i}}{\alpha_{i}}+\left(\frac{\pi_{10}}{\Lambda \mu_{1}}+\Phi_{1}^{+}\frac{\alpha_{1}}{\mu_{1}}\right)\sum_{2}^{n}\frac{\mu_{i}}{\alpha_{i}}-\sum_{2}^{n}\frac{\pi_{i0}}{\Lambda \alpha_{i}},\;or\;\\ 2(1-\Phi_{1}^{+}) & = & 2\Phi_{1}^{+}\frac{\alpha_{1}}{\mu_{1}}\sum_{2}^{n}\frac{\mu_{i}}{\alpha_{i}}+\frac{\pi_{10}}{\Lambda \mu_{1}}\sum_{2}^{n}\frac{\mu_{i}}{\alpha_{i}}-\sum_{2}^{n}\frac{\pi_{i0}}{\Lambda \alpha_{i}}\;,\;that\;yields: \end{array}$$

(A14) $$\begin{array}{ccc} \Phi_{1}^{+} & = & \frac{1+\frac{1}{2\Lambda }\sum_{2}^{n}\left[\frac{\pi_{i0}}{\alpha_{i}}-\frac{\pi_{10}}{\mu_{1}}\frac{\mu_{i}}{\alpha_{i}}\;\right]}{1+\frac{\alpha_{1}}{\mu_{1}}\sum_{2}^{n}\frac{\mu_{i}}{\alpha_{i}}}\;. \end{array}$$

Finally, (A13) and (A14) provide us with the expression

(A15) $$\begin{array}{ccc} \Phi_{i}^{+} & = & \Phi_{1}^{+}\frac{\mu_{i}\alpha_{1}}{\mu_{1}\alpha_{i}}+\frac{\pi_{10}\mu_{i}}{\Lambda \alpha_{i}\mu_{1}}+\Phi_{1}^{+}\frac{\alpha_{1}\mu_{i}}{\alpha_{i}\mu_{1}}-\frac{\pi_{i0}}{\Lambda \alpha_{i}}-\Phi_{i}^{+},\;or\;\\ & = & \Phi_{1}^{+}\frac{\alpha_{1}\mu_{i}}{\alpha_{i}\mu_{1}}+\frac{1}{2\Lambda \alpha_{i}}\;\left[\pi_{10}\frac{\mu_{i}}{\mu_{1}}-\pi_{i0}\right]\;. \end{array}$$

□
